# Supplementary material for: The Chalcone Lonchocarpin Inhibits Wnt/β-Catenin Signaling and Suppresses Colorectal Cancer Proliferation
Source: Cancers (Basel). 2019 Dec 7;11(12):1968. doi: 10.3390/cancers11121968 (PMC6966512; doi:10.3390/cancers11121968)
Supplement: Supplementary file 1 [file cancers-11-01968-s001.pdf]

Supplementary Materials

The Chalcone Lonchocarpin Inhibits Wnt/β-Catenin Signaling and Suppresses Colorectal Cancer Proliferation

Danilo Predes, Luiz F. S. Oliveira, Laís S. S. Ferreira, Lorena A. Maia, João M. A. Delou, Anderson Faletti, Igor Oliveira, Nathalia G. Amado, Alice H. Reis, Carlos A. M. Fraga, Ricardo Kuster, Fabio A. Mendes, Helena L. Borges and Jose G. Abreu

Table S1. Ki67 positive cells means of each treatment group.

Vehicle 50	Vehicle 100	Lonchocarpin 50 mg·kg^{-1}·day^{-1}	Lonchocarpin 100 mg·kg^{-1}·day^{-1}
48.38% [1]	41.03% [1]	40.86% [1]	30.20% [1]
33.57% [1]	33.30% [1]	15.05% [1]	29.16% [1]
44.50% [1]	47.43% [1]	43.22% [1]	31.82% [1]
			23.28% [1]
Mean = 42.15%	Mean = 40.59%	Mean = 33.04%	Mean = 28.62%

[1] The number represents the mean average percentage of Ki67 positive cells of each tested mouse.

Table S2. BrdU positive cell means of each treatment group.

Vehicle 50	Vehicle 100	Lonchocarpin 50 mg·kg^{-1}·day^{-1}	Lonchocarpin 100 mg·kg^{-1}·day^{-1}
29.96% [1]	24.56% [1]	23.47% [1]	11.85% [1]
15.43% [1]	22.01% [1]	6.30% [1]	16.48% [1]
21.53% [1]	19.73% [1]	17.91% [1]	14.14% [1]
			11.98% [1]
Mean = 22.31%	Mean = 22.10%	Mean = 15.89%	Mean = 13.61%

[1] The number represents the mean average percentage of BrdU positive cells of each tested mouse.

A Maternal injection

	Normal	Reduced anterior structures	n
Uninjected	100 %	0 %	25
DMSO	100 %	0 %	15
Ionchocarpin	77 %	23 %	26

B Zigotic injection

	Normal	Enlarged anterior structures	n
Uninjected	100 %	0 %	18
DMSO	100 %	0 %	10
Ionchocarpin	52 %	48 %	29

C Double axis assay

	Normal	Double axis	n
Uninjected	100 %	0 %	32
xWnt8	12 %	88 %	25
xWnt8+DMSO	15 %	85 %	47
xWnt8+Ionchocarpin	32 %	68 %	34

Figure S1. Xenopus laevis embryo assays quantification. (**A**) Quantification of the maternal injection experiment (Figure 3A–D). (**B**) Quantification of the zygotic injection experiment (Figure 3E–H). (**C**) Quantification of the double axis assay (Figure 3I–M,P).

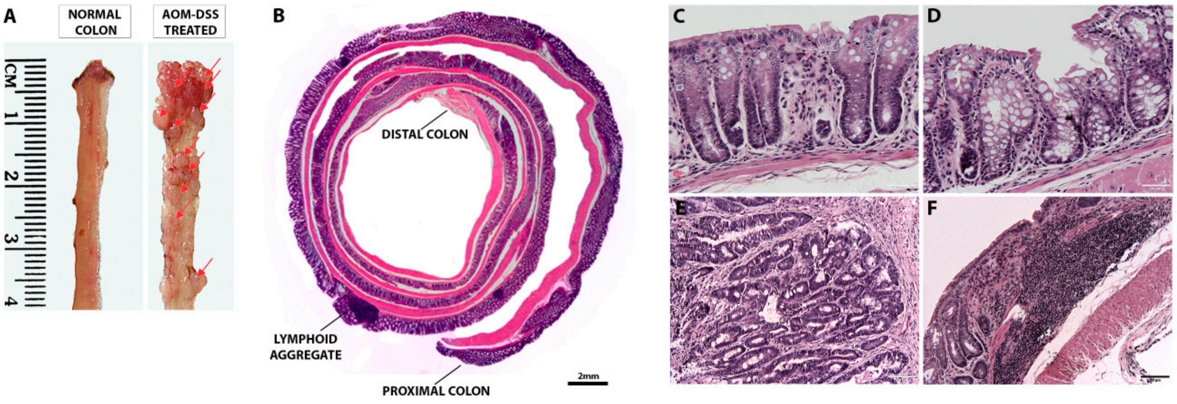

Figure S2. AOM-DSS tumor induction protocol. (**A**,**B**) Colon macroscopic features of the animals. (**B**) Overview of a swiss-rolled normal colon. The most distal portion of the colon is located in the innermost segment of the roller. Scale bar represents 2mm (**C**–**F**) Histopathology of the colon in different sections stained by H&E. (**C**) Normal colon epithelium; (**D**) Aberrant crypt foci; (**E**) Adenoma; (**F**) Leukocyte infiltrate. (**C**–**F**) Magnification: 20x. Scale bar represents 50 µm.

© 2019 by the authors. Licensee MDPI, Basel, Switzerland. This article is an open access article distributed under the terms and conditions of the Creative Commons Attribution (CC BY) license (http://creativecommons.org/licenses/by/4.0/).
